# Analysis of the Enzymatic Properties of a Broad Family of Alanine Aminotransferases

**DOI:** 10.1371/journal.pone.0055032

**Published:** 2013-02-07

**Authors:** Chandra H. McAllister, Michelle Facette, Andrew Holt, Allen G. Good

**Affiliations:** Department of Biological Sciences, University of Alberta, Edmonton, Canada; Lawrence Berkeley National Laboratory, United States of America

## Abstract

Alanine aminotransferase (AlaAT) has been studied in a variety of organisms due to the involvement of this enzyme in mammalian processes such as non-alcoholic hepatocellular damage, and in plant processes such as C_4_ photosynthesis, post-hypoxic stress response and nitrogen use efficiency. To date, very few studies have made direct comparisons of AlaAT enzymes and fewer still have made direct comparisons of this enzyme across a broad spectrum of organisms. In this study we present a direct kinetic comparison of glutamate:pyruvate aminotransferase (GPAT) activity for seven AlaATs and two glutamate:glyoxylate aminotransferases (GGAT), measuring the K_M_ values for the enzymes analyzed. We also demonstrate that recombinant expression of AlaAT enzymes in *Eschericia coli* results in differences in bacterial growth inhibition, supporting previous reports of AlaAT possessing bactericidal properties, attributed to lipopolysaccharide endotoxin recognition and binding. A probable lipopolysaccharide binding region within the AlaAT enzymes, homologous to a region of a lipopolysaccharide binding protein (LBP) in humans, was also identified in this study. The AlaAT enzyme differences identified here indicate that AlaAT homologues have differentiated significantly and the roles these homologues play *in vivo* may also have diverged significantly. Specifically, the differing kinetics of AlaAT enzymes and how this may alter the nitrogen use efficiency in plants is discussed.

## Introduction

Alanine aminotransferase (AlaAT) [E.C. 2.6.1.2], also referred to as glutamate:pyruvate aminotransferase (GPAT), is a pyridoxal-5′-phosphate-dependent (PLP) enzyme that catalyzes the reversible transfer of an amino group from alanine to 2-oxoglutarate to form glutamate and pyruvate [1], [2]. AlaAT is widespread, with homologues in all three biological domains (*Eukarya*, *Archaea* and *Eubacteria*) and functions as a part of several major metabolic pathways. Existing in both the cytosol and the mitochondria, AlaAT plays a critical role in linking carbon and nitrogen metabolism (assimilation and catabolism) within both eukaryotes and prokaryotes [3]. AlaAT is involved in a number of cellular processes including glycolysis, gluconeogenesis, amino acid metabolism [2], hepatocellular damage in mammals [4], photorespiration in plants [5] and nitrogen use efficiency (NUE) in plants, including cereal crops [6], [7], [8]. This latter process is of particular interest, as it has been previously shown that both canola (*Brassica napus*) and rice (*Oryza sativa*) plants over-expressing barley (*Hordeum vulgare*) *AlaAT* (*HvAlaAT*) in a tissue-specific manner have increased NUE under nitrogen (N) limiting conditions, both in controlled environments and field trials for rice [6], [7], [8]. Due to the increased awareness of the detrimental effects of increased N fertilizers in the environment as well as the concerns surrounding increasing world population and subsequent food shortages, the ability to obtain increased yields under limiting N fertilizer conditions is particularly important. Any further understanding of the key enzymes involved in these processes may be of significance in additional improvements in NUE [9], [10], [11].

To date, AlaAT enzymes and their kinetics have been characterized in a variety of species. However most work on this enzyme has focused on the medical implications of the AlaAT isoforms found in humans (HsAlaAT) [12]. Increases of both HsAlaAT1 (cytoplasmic) and HsAlaAT2 (mitochondrial) in mammalian serum samples have shown to be reliable indicators of liver damage, muscle damage and celiac disease [13]. Moreover, significant increases in activity of mouse (*Mus musculus*) mitochondrial AlaAT (MmAlaAT2), but not mouse cytoplasmic AlaAT (MmAlaAT1) in fatty livers of obese mice, indicate possible differences in the roles/effects these two isozymes have in the cell. The evolution of differences in the kinetics of various isozymes of AlaAT would be driven in part by the distinct cellular roles these isozymes play [4].

Good *et al.*, [7] and Shrawat *et al*., [8] observed a nitrogen use efficient (NUE) phenotype in plants with over-expression of the HvAlaAT enzyme in canola and rice, respectively, using a tissue specific promoter. However, the specific basis for this phenotype remains unclear and there is a continued effort to understand the intracellular mechanisms which cause this phenotype. One question of particular interest is whether different AlaAT enzyme isoforms have different kinetics and if so, could these different isoforms favor an NUE phenotype? More specifically, are there optimal kinetic properties of AlaAT which can produce an increase in NUE when expressed within plants? Given that the previous NUE phenotypes were observed in canola and rice utilizing a promoter which increased expression in the roots [14], the benefits of targeting the expression of a gene of interest to a particular tissue have become clear. For example, genes involved in producing modified oils are usually expressed with a seed specific promoter [15]. What has been less studied is the importance of choosing an enzyme that works with optimal efficiency in the appropriate environment and tissue. The importance of studying enzyme variants is illustrated by the example of *Golden Rice*. Development of this rice involved the insertion of a daffodil phytoene synthase (*psy*) gene for the efficient production of ß-carotene, a product used to synthesize Vitamin A [16]. A number of *psy* genes were analyzed in order to determine which produced the highest levels of ß-carotene, and which variant was rate-limiting; further analysis revealed that an even more efficient *psy* gene may exist in maize (“Golden Rice 2”) [16]. Therefore, identifying enzyme variants that overcome a metabolic bottle-neck could prove to be an effective strategy for trait improvement.

To investigate further the basis for an increased NUE phenotype, we chose to evaluate different enzyme variants of AlaAT with a view to using these variants to gain insights into the underlying metabolic changes that affect NUE in plants. Because AlaAT has an equilibrium constant near one, the reaction of this enzyme *in vivo* will be driven by substrate concentrations [17]. Therefore, it follows that an AlaAT homologue with increased specificity or different kinetic properties could allow for increased NUE properties in a plant system. This approach was recently taken by Duff *et al*., who examined the kinetic properties and crystal structure of different AlaAT enzyme variants [17]. Here we present a kinetic comparison of AlaAT enzymes from a broader variety of organisms, placing emphasis on the difference in K_M_ values between homologues enzymes instead of the specific activity of the enzyme which has been analyzed elsewhere [17]. Furthermore, AlaAT enzymes used in this analysis were not tagged as has been done previously, which can affect enzyme activity. Finally, only L-amino acid enzymes were used in this analysis given that many plant pathways are L-enantiomer stereospecific, including shikimate, aspartate, pyruvate and glutamate [18], and that a very small percent (∼0.5–3) of the total amino acids within many plants are not of the L-type [19]. To our knowledge, this is the most comprehensive kinetic analysis of AlaAT homologues. We show that AlaAT homologues and two glutamate: aminotransferase (GGAT) enzymes (which have secondary glutamate:pyruvate aminotransferase activity) have relative K_M_ values for co-substrates that indicate that *in vivo* the rate and direction of the reaction catalyzed by each enzyme, under similar substrate concentrations, may differ dramatically. These results reaffirm the results obtained by Duff *et al.,* [17] for some of the variants tested with the addition of K_M_ values for eight AlaAT’s not studied previously. The effects of various enzymes with diverging kinetic behaviours were also assessed for functional consequences in *E. coli* under different environmental conditions.

## Results and Discussion

### Homologous AlaAT Primary Sequence Comparison

**Figure 1:**
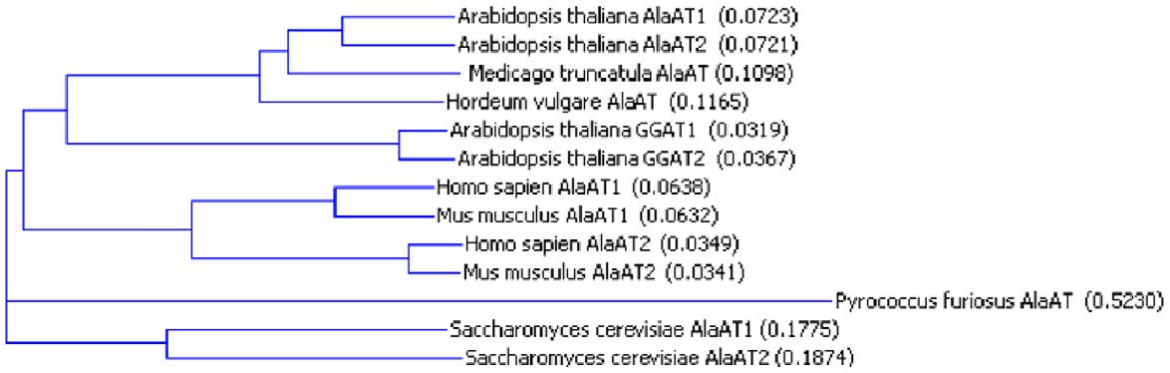
Phylogenetic dendrogram of eleven AlaAT enzymes and two GGAT enzymes. A phylogenetic dendrogram was constructed using neighbour joining (NJ) based on amino acid sequence similarity using Vector NTI Advance v. 11.0, AlignX software. The sequences used to construct the dendrogram were obtained from the NCBI database except that of Medicago which was obtained courtesy of Anis Limami at the Université d' Angers.

Glutamate:pyruvate aminotransferases (AlaAT/GPAT) and glutamate:glyoxylate aminotransferases (GGAT) are subgroup I aminotransferases, containing eleven invariant residues essential for binding the coenzyme PLP and for stabilizing the enzyme:substrate transition state [1], [5]. Both AlaAT and GGAT enzymes share similar primary and secondary structures, as well as hydropathy with other subgroup I aminotransferases such as aspartate aminotransferase and tyrosine aminotransferase [1]. All 13 enzymes examined in this study showed this conservation and maintained the 11 invariant residues previously defined for subgroup I aminotransferases (see Ward *et al.,* 2000 [20]) (Figure S1). Primary sequence analysis (Figure 1) indicates that of the sequences studied, *P. furiosus* (PfAlaAT) is the most divergent, which is not surprising considering this was the only non-eukaryotic sequence examined. Interestingly, the protein sequences of AtGGAT1 and AtGGAT2 are more similar to plant AlaAT enzymes than are mammalian and archaean AlaAT enzymes to plant homologues, even though GGAT enzymes are capable of both glutamate:glyoxylate and glutamate:pyruvate aminotransferase reactions [5]. It appears that the kinetic differences identified here are due to differences in non-conserved residues which may cause changes in substrate binding affinity and/or catalytic rate, perhaps as a result of changes in enzyme folding.

### AlaAT Enzymes have Varying Substrate K_M_ Values

K_M_ values from eight AlaAT and two GGAT enzymes are compared in Table 1. Although K_M_ values for several of the enzymes analyzed here have been reported previously, our study facilitates a comparison of data obtained with a single assay system. To date, most studies on AlaAT have been organism and tissue-specific, focusing on a single enzyme or isozymes, making comparisons between AlaAT enzymes from different species difficult. Comparisons between enzymes have also been limited due to purification and expression differences, as in the case of AtGGAT1. The AlaAT activity of this enzyme has been examined by purification of this protein from both shoot tissues [21] and recombinant *E. coli* [5]. These differences in enzyme source and purification procedure can manifest as alterations in enzymatic kinetic behaviour, compounded by differences in the assay conditions used during kinetic analyses [20], [22], [23]. Other errors may result from the presence of homologous proteins which were not separated from the AlaAT of interest during purification, due to similar biochemical properties and increases in activity from environmental changes (e.g. greening of leaves) [24]. Due to these confounding factors, kinetic data obtained with enzymes from various sources cannot be reliably compared across different studies (Table 2).

**Table 1:** Summary of kinetic assay results.

Enzyme	Alanine		2-oxoglutarate		Glutamate		Pyruvate	
	K_M_ (mM)	SE K_M_	K_M_ (mM)	SE K_M_	K_M_ (mM)	SE K_M_	K_M_ (mM)	SE K_M_
**AtAlaAT1**	2.4	±0.5	0.1	±0.0	2.5	±0.4	0.1	±0.0
**AtAlaAT2**	10.4	±1.2	1.0	±0.1	4.9	±1.1	5.1	±0.9
**AtGGAT1**	1.9	±0.5	0.2	±0.1	0.2	±0.1	0.6	±0.1
**AtGGAT2**	1.2	±0.1	8.8	±1.8	0.2	±0.1	18.5	±2.7
**HvAlaAT**	5.6	±0.6	0.2	±0.0	4.9	±0.5	0.1	±0.0
**MtAlaAT1**	1.0	±0.2	0.2	±0.0	0.1	±0.0	18.6	±2.7
**MtAlaAT2**	1.5	±0.2	0.3	±0.0	0.3	±0.1	18.0	±1.9
**MmAlaAT1**	26.5	±2.3	0.1	±0.1	13.0	±2.4	12.5	±2.0
**PfAlaAT**	4.0	±0.5	0.02	±0.0	0.9	±0.1	16.5	±2.2
**ScAlaAT1**	0.3	±0.1	0.5	±0.1	0.7	±0.1	11.0	±1.7

K_M_ values are shown for each substrate, for each of the ten enzymes examined. Kinetic values represent the average of three independent trials. The correlation coefficient (r^2^) was >0.80 for all trials, except AtGGAT1 glutamate, AtGGAT2 glutamate and MmAlaAT1 pyruvate. Raw data are plotted in Figure S2.

**Table 2:** Comparison of K_M_ values for AlaAT and GGAT enzymes.

Organism	K_M_ (mM)				Previous K_M_ (mM)				Source of AlaAT	Reference
	Alanine	2-Oxoglutarate	Pyruvate	Glutamate	Alanine	2-Oxoglutarate	Pyruvate	Glutamate		
***Arabidopsis*** ** AlaAT1**	2.4	0.1	0.1	2.5	1.5	0.2	NA	NA	*A. thaliana* leaves (protein purified)	Wiśniewski *et al*., (2006)
**Barley AlaAT**	5.6	0.2	0.1	4.9	17	5	0.1	1	Barley roots (protein purified)	Good and Muench (1992)
***Arabidopsis*** ** GGAT1**	1.9	0.2	0.6	0.2	4.8	0.3	0.3	2.0	*E. coli* (recombinant expression)	Liepman and Olsen (2003)
					NA	NA	NA	1.2	*A. thaliana* leaves (protein purified)	Wiśniewski *et al*., (2006)
***Arabidopsis*** ** GGAT2**	1.2	8.8	18.5	0.2	3.6	0.5	0.4	3.3	*E. coli* (recombinant expression)	Liepman and Olsen (2003)
***Pyrococcus furiosus*** ** AlaAT**	4.0	0.02	16.5	0.9	3.2	1.1	4.7	5.6	*E. coli* (recombinant expression)	Ward *et al*., (2000)

K_M_ values obtained during this study were compared with previous K_M_ values from various published sources. Discrepancies between kinetic values can be attributed partially to differences in procedure as well as differences in original data collection. Nevertheless, significant differences in K_M_ values for all enzymes are evident. Variation amongst results highlights the benefit of using one system and one procedure when comparing kinetic data from several different enzymes. (NA = no data available from previous studies.).

K_M_ values reported here indicate significant diversity between the different enzymes for the same substrates. Between AtAlaAT1 (cytoplasmic) and AtAlaAT2 (mitochondrial) the minimal K_M_ discrepancy between substrates was reported for glutamate, with an approximate two-fold increase in K_M_ (2.5 mM to 4.9 mM respectively) (Table 1). The K_M_ values obtained from both *M. truncatula* proteins were similar for all substrates, the greatest difference being seen for the substrate glutamate, with a three-fold change in K_M_ (0.1 mM for MtAlaAT1, 0.3 mM for MtAlaAT2). The largest difference between substrates for a single enzyme was seen for PfAlaAT. For this enzyme, there was an 825-fold difference between K_M_ values for 2-oxoglutarate and pyruvate (0.02 mM and 16.5 mM respectively). The second largest difference in K_M_ values for a given enzyme between substrates was seen for MmAlaAT, with a 265-fold difference (seen between the K_M_ values for alanine and 2-oxoglutarate, 26.5 mM and 0.1 mM, respectively). No groupings or patterns could be established among the K_M_ values obtained, and relative differences were not consistent for a single enzyme and multiple substrates, or for the K_M_ values of multiple enzymes for a single substrate. K_M_ values for 2-oxoglutarate appeared to be reasonably constant (difference in K_M_ values of 8.78 mM) with AtGGAT2 having a K_M_ of 8.8 mM. The next largest value belonged to AtAlaAT2, with a K_M_ for 2-oxoglutarate of 1.0 mM. The range of K_M_ values for alanine, pyruvate and glutamate were much greater (differences in K_M_s of 26.2 mM, 12.9 mM and 18.5 mM respectively).

The K_M_ values reported here for HvAlaAT and ScAlaAT1 share some similarity to those recently reported by Duff *et al.,* [17], with the largest difference between values being the K_M_ for ScAlaAT1 and the substrate pyruvate, here reported as a K_M_ of 11.0 and previously reported as a K_M_ of 0.4, a 27.5 fold difference. All other K_M_’s for the remaining substrates alanine, 2-oxoglutarate and glutamate showed lower fold differences when K_M_ values for ScAlaAT were compared, 12, 2.5 and 2.9 respectively. The K_M_ values reported for HvAlaAT did not show as great a deviation between studies for the various substrates with, 6.7, 1.1, 9.1 and 2.1 fold differences for alanine, 2-oxoglutarate, glutamate and pyruvate respectively. These discrepancies in K_M_ values could be the result of numerous protocol differences as outlined above, and re-emphasize the importance of obtaining enzymatic data from a single source for the purpose of direct comparisons.

V_max_ values for all enzymes assayed are presented in Table S1. Since enzyme fractions were not purified, the concentrations of the enzymes used, and thus catalytic rate constants are unknown, therefore the usefulness of V_max_ values in making meaningful comparisons between different enzymes is diminished. Purification of individual proteins in order to establish enzyme concentrations, thereby allowing determination of k_cat_ and comparison of V_max,_ was not done due to the absence of an antibody that would specifically bind each of the different variants for purification purposes. Furthermore, enzymes were not tagged with either His or Myc-C sequences since such alterations may affect enzyme kinetics. Given that these were recombinant proteins expressed in a bacterial system, protein folding may have been altered affecting kinetic results. Since whole protein fractions were utilized during this study, the possibility that enzyme inhibitors were present or that non-AlaAT transaminase activities may have contributed to substrate turnover as well as NAD/NADH concentrations and influenced calculated kinetic constants must be considered.

It is therefore of interest to determine the effects on NUE phenotypes of AlaAT enzymes that display kinetics similar to those of HvAlaAT, compared with enzymes that have very different characteristics. Based on the results from the kinetic assays, AtAlaAT1 appears to be most similar to HvAlaAT. Both AtAlaAT enzymes have higher K_M_ values for glutamate and alanine, and lower K_M_ values for 2-oxoglutarate and pyruvate, compared with HvAlaAT. MmAlaAT1 has rather different K_M_ values, raising the possibility of a distinct *in vivo* role(s). Compared with other AlaATs examined, this enzyme had the highest K_M_ values for both alanine (26.5 mM) and glutamate (13.0 mM).

It is difficult to extrapolate *in vitro* kinetic data and predict the consequences of altered substrate K_M_ values, without knowing the cellular concentrations of substrates under various environmental conditions. For example, while MmAlaAT appears to display very different kinetic behavior compared with HvAlaAT, *in vivo* analysis will be needed in order to verify whether or not such differences have any effect on plant phenotype when the gene is ectopically expressed. Alanine and glutamic acid concentrations in rice seeds show average millimolar amounts of alanine as ∼0.57 mM and glutamic acid as ∼1.53 mM, with vitamin B_6_, a precursor to the AlaAT cofactor PLP, at ∼0.72 mM [25]. Other studies conducted by Narsai *et al.,* [26] show that in rice seedlings these concentrations are altered during the growth and development of the plant and are approximately 0.09 mM for glutamate, 2.27 mM for alanine, 0.79 mM for pyruvate and 0.65 mM for 2-oxoglutarate. The changes in AlaAT substrate concentrations during different phases of plant growth and in various cellular tissues and organs has also been documented elsewhere [27]. Aminotransferase enzymes with overlapping functions have also been observed in a number of organisms, including *E. coli* [28], *P. furiosus* [20] and plants [17], [29]. The effect of any of these enzymes on nitrogen uptake or metabolism can only be speculated upon and would require whole plants studies and *in vivo* analysis which are currently underway. Analysis of the kinetics of aspartate aminotransferase (AspAT) from higher plants has also been carried out recently with similar intentions of crop improvement [30].

### AlaAT Homologues Differ in their Ability to Reduce Growth Rate of Gram Negative Bacteria

**Figure 2:**
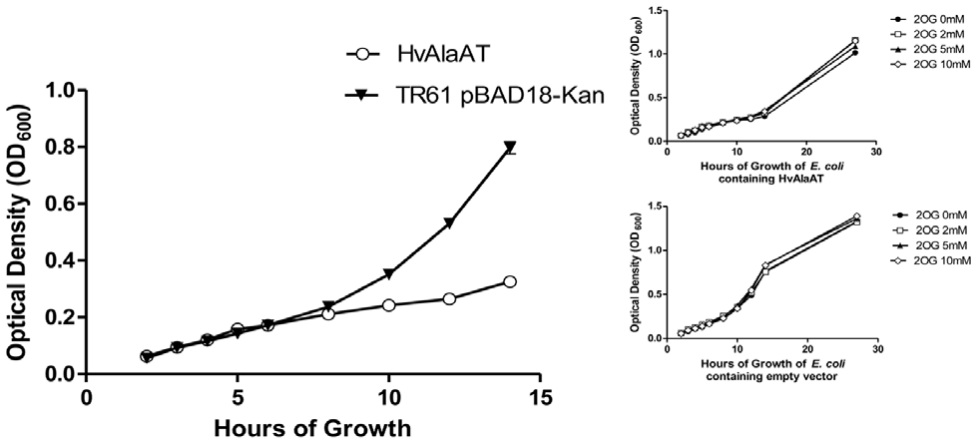
Effect of 2-oxoglutarate on growth rate of *E. coli* expressing *HvAlaAT*. Growth of TR61 cells containing either pBAD:HvAlaAT or TR61 cells containing an empty pBAD18-Kan vector in modified M63 medium. *E. coli* containing the two constructs grown in media containing 0 mM, 2 mM, 5 mM or 10 mM 2-oxoglutarate (2OG), pH 8.0 (inset).

**Figure 3:**
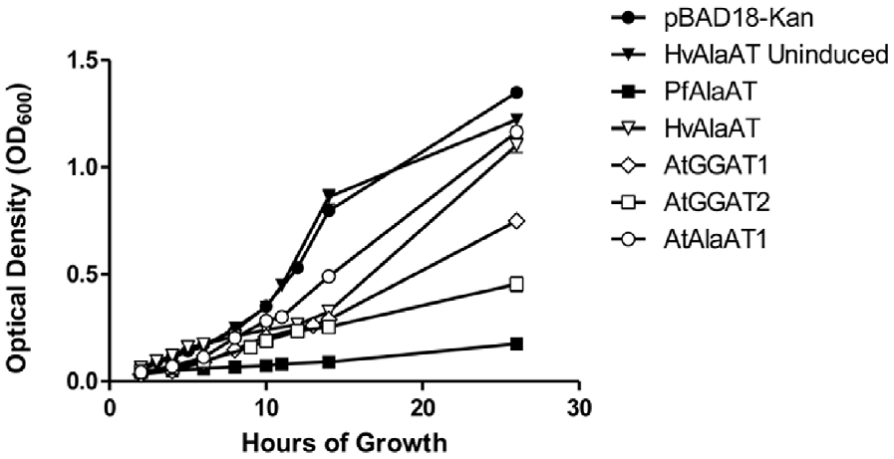
The average growth of *E. coli* containing various AlaAT and GGAT enzymes. TR61 *E. coli* were cultured in a M63 revised minimal medium and expression of AlaAT or GGAT was induced by the addition of arabinose. The average optical density at 600 nm was measured and recorded over a 26 hour time period and is representative of trials done in triplicate. Error bars show standard error, where they exceed symbol size. TR61 cells containing no pBAD vector, cells containing a pBAD vector with no *AlaAT* or *GGAT* insert and uninduced TR61 cells containing a pBAD::HvAlaAT enzyme were utilized as controls. *E. coli* expressing *AtAlaAT2*, *MtAlaAT*, *ScAlaAT* and *MmAlaAT* showed changes in optical density over the 26 hour time period similar to that seen by *Arabidopsis thaliana AlaAT1* expressing cells.

As an initial screen to determine if the presence of a specific AlaAT variant in *E*. *coli* had a significant effect on the bacteria’s ability to utilize specific substrates, *E. coli* expressing the various AlaAT enzymes from plasmid constructs were grown in modified M63 medium supplemented with various concentrations of 2-oxoglutarate, with ammonium as the nitrogen source. It was speculated that 2-oxoglutarate might have an effect on the growth rates of *E. coli* due to its central role in linking both carbon and nitrogen metabolism in bacteria [31], [32], [33], [34]. Furthermore, excess 2-oxoglutarate in the growth medium may have a significant impact on AlaAT enzymes with lower K_M_ values for both alanine and 2-oxoglutarate, if substrate k_cat_ values of these enzymes are not also lower and assuming that substrates are present at sub-saturating concentrations. It was hoped that changes in the availability of AlaAT substrate(s) (2-oxoglutarate) during growth of *E. coli* over-expressing various AlaAT homologues would allow for differentiation of homologous enzymes in terms of substrate usage, manifest phenotypically as changes in rates of growth. For the reasons outlined below, we were unable to characterize the transgenic *E. coli* in terms of changes to available substrate concentrations however the results indicate that AlaAT may maintain novel functions within *Eukarya*, *Archaea* and *Eubacteria.* Whether these functions play a role in plant NUE has yet to be explored. Ultimately, no difference in growth rate of *E. coli* containing HvAlaAT was observed when exposed to concentrations of 2-oxoglutarate (Figure 2). However we did observe a slow growth phenotype in all *E. coli* cultures expressing the various *AlaAT* constructs (Figure 2 and Figure 3).

**Figure 4:**
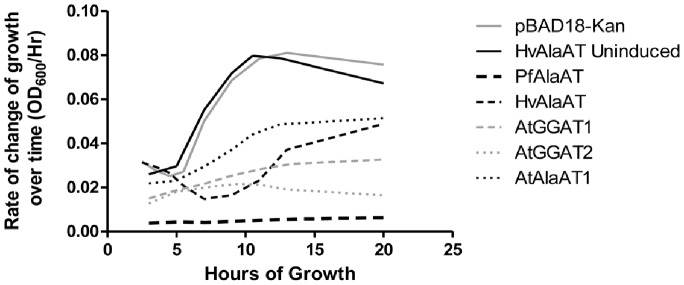
The rate of change of the growth over time (OD_600_/Hrs) of *E. coli* strains containing various alanine aminotransferase enzymes. The smooth first derivative of each time trial in Figure 4 was determined, indicating the change in the growth rate of the bacteria containing each of the different AlaATs and the controls over time. *E. coli* expressing *AtAlaAT2, MtAlaAT*, *ScAlaAT1* and *MmAlaAT1* showed changes in the rate of growth over the 26 hour time period similar to that seen by *Arabidopsis thaliana AlaAT1* expressing cells.

Expression of recombinant *AlaAT* from *B. japonicus* (*AmphiALT*), in gram-negative *E. coli* has been shown to cause cell lysis through lipopolysaccharide (LPS) binding [35]. Jing and Zhang (2011) [35] observed that AmphiALT was able to bind and lyse gram-negative but not gram-positive cells; binding was reported to be specific to the LPS region. From these results it was suggested that AlaAT may also be involved in the acute phase response, particularly in liver tissues. Our study supports this view, indicating that recombinant expression of AlaAT in *E. coli* inhibits growth, and decreases cell growth rates when grown in minimal medium (Figure 3 and Figure 4). Due to the absence of antibodies for all AlaAT enzymes and thus an inability to purify individual AlaATs, direct binding of *E. coli* LPS to the various AlaAT enzymes was not examined. However, slow growth phenotypes similar to those described previously in the presence of AlaAT were observed, leading to the conclusion that all of the AlaAT enzymes studied exhibit some bactericidal activity similar to *AmphiALT* from *B. japonicus*, and providing evidence for the conservation of AlaAT bactericidal properties. In order to clarify, the growth curves of only five of the ten *AlaATs* expressed in *E. coli* are shown in Figure 3. *E. coli* cells expressing *AtAlaAT2*, *MtAlaAT*, *ScAlaAT* and *MmAlaAT1* showed growth curves similar to those obtained with *AtAlaAT1*.

Among the ten enzymes assayed, inhibition of growth rate varied significantly. PfAlaAT showed the greatest effect on growth rate over time, seen most clearly in Figure 4, when the first derivative of each sample is determined. Both AtGGAT2 and AtGGAT1 also show dramatic decreases in growth rate over time (Figure 4). Comparison of kinetic constants for a particular enzyme with growth assay results has not provided any insights into the reasons for these differences in growth rates. Also interesting was the observed change in the growth rate of the bacteria containing the HvAlaAT over time (Figure 4). While a constant or slight increase in growth rate was observed with the majority of proteins assayed, the over-expression of HvAlaAT resulted in a substantial decrease in growth rate starting at approximately 2 hours and continuing until approximately 7 hrs, at which point growth rate once again began to increase.

**Figure 5:**
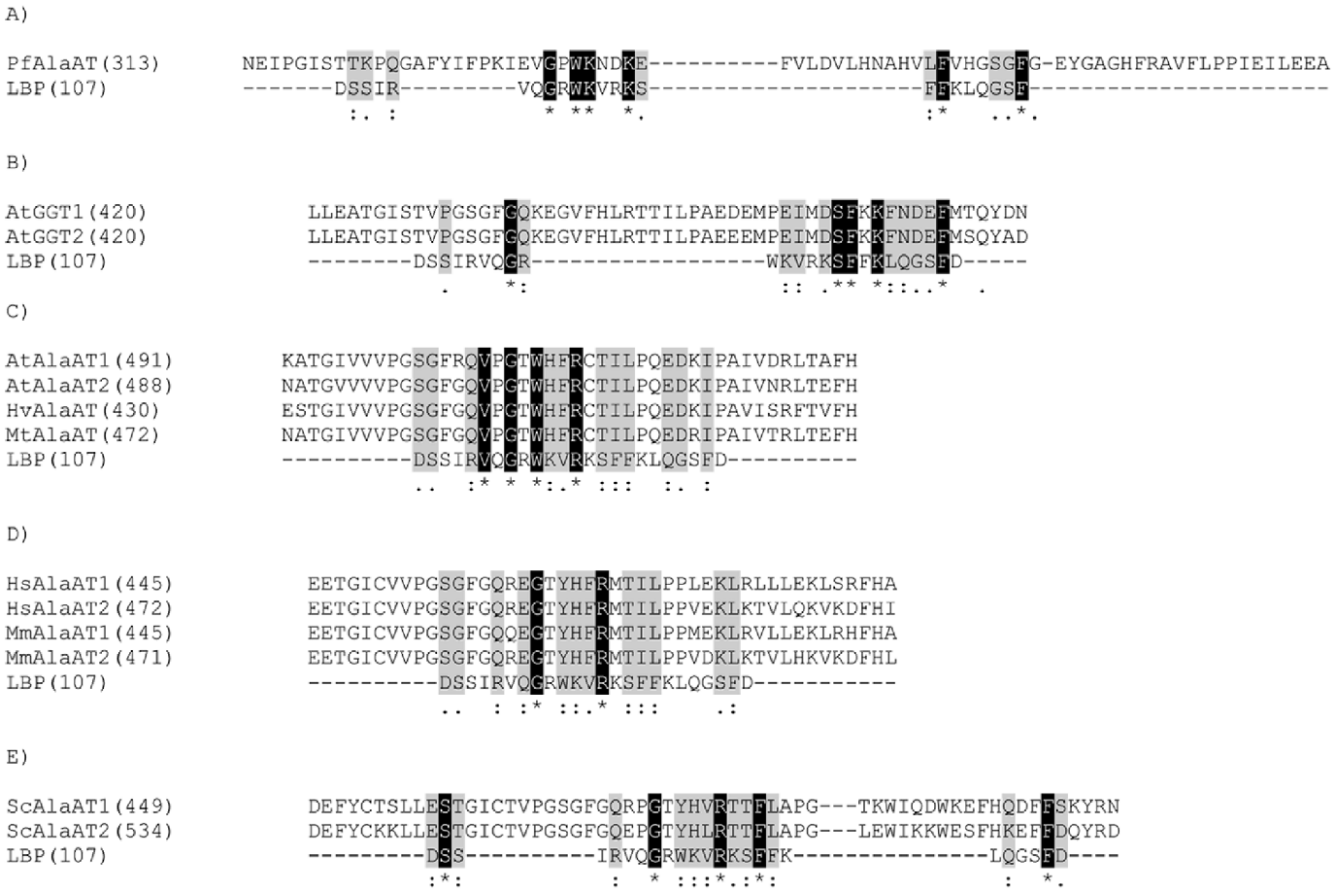
Comparison of AlaAT putative endotoxin binding regions. Primary sequence comparison of AlaAT homologues to the known endotoxin binding region of human LBP (A–E) using the ClustalW alignment program. Identical residues are highlighted in black with white text while conservative and semi-conservative residues are highlighted in grey with black text. (“*” indicate identical residues: “:” indicates conservative substitutions; “.” indicates semi-conservative substitution.

To investigate further the bactericidal activity of AlaAT, the primary structure of each enzyme was analyzed for a conserved endotoxin binding region. Through analysis of the known LPS region from human lipopolysaccharide binding protein (LBP) [36], a similar conserved region was found to various extents in all AlaAT enzymes (Figure 5). PfAlaAT shows the greatest conservation of the LPS binding region, with the highest number of residues conserved, and a percent identity of 26% (Figure 5A). PfAlaAT was also the most effective isoform inhibiting growth of *E. coli* (Figure 4). AtGGAT1 and AtGGAT2 also show a high degree of conservation of identical residues, with percent identities of 22% (Figure 5B). All sequences show a high degree of conservative and semi-conservative sequence similarity, for example with AtAlaAT1, AtAlaAT2, MtAlaAT and HvAlaAT (Figure 5C), HsAlaAT1, HsAlaAT2, MmAlaAT1 and MmAlaAT2 (Figure 5D), and ScAlaAT1 and ScAlaAT2 (Figure 5E). However the ability of these enzymes to bind the LPS region of *E. coli* and inhibit growth was not as pronounced as that observed for PfAlaAT, AtGGAT1 and AtGGAT2, perhaps demonstrating the importance of a high level of conservation of sequences/residues at these sites. The possibility that other conserved endotoxin binding regions may exist to varying degrees in the AlaAT homologues analyzed should also be considered as this could also contribute to the variation of growth inhibition observed. Given the evidence that AlaAT enzymes may be playing a role in acute phase response to bacterial infections *in vivo* [35], determining how these differences affect, or are affected by concentration-dependent binding of other molecules within the cell will be important to develop a more complete understanding of enzyme function.

### Conclusion

A kinetic examination of the enzyme alanine aminotransferase is of interest for a number of reasons. AlaAT has been shown to be involved in stress responses in numerous plants, including cereal crops [2], [22], [37], while in mammals, this enzyme is used as an indicator of non-alcoholic hepatocellular liver damage [13] and may be involved in immune system acute phase response [35]. Recently, AlaAT has been shown to increase NUE in cereal crops when over-expressed with a tissue specific promoter [6], [8]. The kinetic results presented here indicate that catalytic properties between AlaAT homologues differ considerably. Our data reveal that when AlaAT is over-expressed in plant systems, various phenotypic results with respect to changes in NUE may be observed. Previous analysis in *Brassica napus* and rice has already determined the importance of using a tissue-specific promoter in obtaining an NUE phenotype [6], [7], [8], [38], [39]. Preliminary studies in our group indicate that *Arabidopsis thaliana* over-expressing several of the different AlaAT enzymes with differing kinetics can produce novel phenotypes. The knowledge of enzyme variants gained here, as well as prior knowledge of promoters provides a platform for future NUE studies and the improvement of crop nutrient utilization.

## Materials and Methods

### pBAD18-Kan:*AlaAT* Constructs

The alanine aminotransferase enzymes assayed were chosen based on differences in their amino acid sequence (Figure S1) and the availability of a cloned gene. Barley alanine aminotransferase *(Hordeum vulgare, HvAlaAT*) (GenBank accession no. Z26322) was obtained from a cDNA originally used in our over-expression studies [8] and was described by Muench and Good (1994) [40]. Both *Medicago truncatula* cDNA sequences were obtained from Anis Limami at the Université d' Angers [41]. Although only one naturally-occurring mitochondrial *Medicago truncatula* alanine aminotransferase (*MtAlaAT*) has been described (*Medicago truncatula* genome sequencing resources, Medtr8g023140), none of the sequences received were identical to the expected sequence, and so two of the most similar sequences were chosen to be expressed and analyzed. MtAlaAT1 contained the point mutation I144V while MtAlaAT2 contained the point mutation F177S. *Pyrococcus furiosus AlaAT* (*PfAlaAT*) (GenBank accession no. NP_579226) was amplified from ATCC gDNA (DSM 3638). *Arabidopsis thaliana AlaAT1* (*AtAlaAT1*) (TAIR reference no. AtG17290) and *AlaAT2* (*AtAlaAT2*) (TAIR reference no. At1G72330) sequences were obtained from Yo Miyashita [2]. Yeast (*Saccharomyces cerevisiae*) sequences (*ScAlaAT1,* GenBank accession no. NP_013190 and *ScAlaAT2,* Genbank accession no. NP_010396) were amplified from ATCC gDNA (S288C [MUCL 38902]). *Arabidopsis thaliana AtGGAT1* (TAIR reference no. At1G23310) and *AtGGAT2* (TAIR reference no. At1G70580) (*glutamate:glyoxylate aminotransferase*) sequences were obtained from Laura Olsen at the University of Michigan [5]. Mouse (*Mus musculus*) *MmAlaAT1* (GenBank accession no. NP_877957) and *MmAlaAT2* (GenBank accession no. NP_776291) sequences were acquired from Rong ze Yang at the University of Maryland [13], as were those for human (*Homo sapien*) *HsAlaAT1* (GenBank accession no. NP_005300) and *HsAlaAT2* (GenBank accession no. NP_597700). All genes were sequenced and the primers used for sequencing are listed in Table S2. When available, sequences were compared with BLAST results from the NCBI nucleotide database.

All of the sequences were cloned into the *E. coli* expression vector pBAD18-Kan using primers containing approximately 22 bps of *AlaAT* specific gene sequence and a restriction digest cut site at the 5′ end (Table S2). Forward primers contained cut sites for either Kpn1 or Sac1, while Xba1 cut sites were used for the reverse primers. These constructs were transformed into *E. coli* TR61 strain K-12 cells and used for *AlaAT* expression, activity and kinetic assays. TR61 cells are derived from the *E. coli* K-12 strain MC4100, containing a *lac* reporter gene on a lambda phage insertion and a Tn10 insertion conferring resistance to the sugar arabinose, and were a gift from Tracy Ravio at the University of Alberta. TR61 cells are a previously unpublished *de novo* cell line and permission for their use was granted by Tracy Ravio and the Biosafety Committee, University of Alberta.

### Analysis of AlaAT Primary Structure

Thirteen enzymes with AlaAT activity were obtained for expression studies and kinetic analysis and the amino acid sequences were compared using ClustalW software (full primary sequence comparison is provided in Figure S1). The *Medicago truncatula* sequence utilized for this analysis was obtained from Anis Limami at the Université d' Angers [41]. Vector NTI Advance v. 11.0 software was used to construct a dendrogram (Figure 1). For both ClustalW and Vector NTI analysis, a gap open penalty of 10 and a gap extension penalty of 0.05 were used. The percent identity of amino acid sequences was defined by dividing the number of identical residues by the number of amino acids in the shortest sequence; gaps were not taken into account.

### Induction of AlaAT Constructs in *E. coli*

Five hundred µL of *E. coli* TR61 overnight cultures containing the various *AlaAT* constructs were added to 45 mL LB and grown at 37°C, to an OD_600_ of 0.5–0.75, at which point 20 mL of culture was added separately to two flasks, one of which received arabinose to a final concentration of 0.2%. Both induced and uninduced cultures were incubated, shaking at 37°C for an additional 4 hrs. Induced and uninduced cultures were harvested by centrifugation after 3–4 hrs, washed a single time with STE buffer (10 mM Tris-HCl pH = 7.5, 1 mM EDTA, 150 mM NaCl), and bacterial pellets were flash frozen and stored at −80°C. Cultures were stored at −80°C for no more than 2 weeks prior to protein extraction.

### Protein Extraction

Whole protein fractions were extracted using a freeze-thaw method. Cells were re-suspended in 2 mL protein extraction buffer (100 mM Tris-HCl pH = 7.5, 5 mM EDTA) containing 1 mM DTT and 1 mM PMSF. Ten µL of 10% Triton-X100 and 10 µL of 1 mg/mL lysozyme (Sigma, L-6876) were added to resuspended cells and incubated at room temperature for 15–30 min. Protein was extracted through six cycles of freeze-thaw using liquid nitrogen. Samples were then centrifuged and the supernatant from each cell fraction was removed and applied to a PD-10 desalting column (GE Healthcare, Sephadex G-25 M, PD-10 Columns). The eluate from these columns was used for both AlaAT activity measurements and kinetics assays.

### Activity Assays

Extracts were tested for glutamate:pyruvate aminotransferase (GPAT) activity prior to kinetic assays to ensure the induction of *AlaAT* expression. Varying dilutions of the AlaAT/GGAT protein extractions were assayed alongside the uninduced protein fractions to ensure the presence and activity of the AlaAT/GGAT enzymes. Leaky expression of AlaAT in the uninduced protein fractions was regularly observed, but at very low levels. Activity assays were also conducted in order to determine the optimal degree of dilution of the enzyme necessary for kinetic assays; these typically ranged between 30X–40X. Activity assays were prepared and carried out at 20°C in the direction alanine and 2-oxoglutarate to pyruvate and glutamate. Activity of AlaAT enzyme was determined using a continuous coupled reaction catalyzed by lactate dehydrogenase (LDH, Sigma, L-2518), with the change in absorbance associated with generation of NAD^+^ from NADH monitored at 340 nm. Assays, done in 96 well microplates (UV-Star, VWR, 82050-788), were initiated by the addition of 10 µL of protein extracts, including the undiluted fraction (maximal activity of sample before dilution) and the undiluted uninduced (negative control) protein samples, to 290 µL of an AlaAT assay buffer (0.1 M Tris-HCl pH 8.0 at 20°C, 10 mM 2-oxoglutarate, 670 mM alanine, 0.27 mg ml^−1^ NADH, 0.36 U LDH, 10 µM pyridoxal-5′-phosphate (PLP)) such that the final reaction volume was 300 µL per well. The change in absorbance at 340 nm was monitored continuously for 10 min in a SpectraMax Plus absorbance plate reader (Molecular Devices, Sunnyvale, CA).

### Kinetic Assays

Kinetic assays were run for both sets of AlaAT substrates, with the concentration of one substrate varied while the other was held constant at a saturating concentration, for 10 of the 13 constructs. Kinetic data were not obtained for MmAlaAT2-pBAD18-Kan, ScAlaAT2-pBAD18-Kan, HsAlaAT1-pBAD18-Kan, or HsAlaAT2-pBAD18-Kan as activity of these constructs was not detected in initial assays. We believe that this inability to detect activity was the result of inclusion body formation with these proteins in *E. coli*.

Enzyme activity at each substrate concentration was assayed at 20°C in triplicate, over a concentration range(≈ 0.3×K_M_–8×K_M_) chosen based on previously published values and preliminary kinetic assessments. To each well, 10 µL of diluted AlaAT protein sample were added, along with 20 or 50 µL of substrate, and kinetic assay buffer to a final volume of 300 µL. When AlaAT activity was measured in the direction alanine to pyruvate, the kinetic assay buffer consisted of either alanine (100 mM) or 2-oxoglutarate (10 mM), 0.1 M Tris-HCl, pH 8.0, 0.27 mg ml^−1^ NADH, 10 µM PLP and 0.36 U LDH. When activity in the direction of pyruvate to alanine was measured, the assay buffer contained either pyruvate (10 mM) or glutamate (50 mM), 0.1 M Tris-HCl, pH 8.0, 100 mM NH_4_Cl, 0.27 mg ml^−1^ NADH, 10 µM PLP and 1.14 U glutamate dehydrogenase (GDH, Sigma, G-2501). The change in absorbance at 340 nm was monitored continuously for 6–10 min in a SpectraMax Plus platereader. The initial pseudo-linear portion of each absorbance-time plot was analyzed by linear regression (SoftMax Pro v. 3.0) to obtain initial rates. Thereafter, plots of initial rate *versus* [substrate] were fitted to the Michaelis-Menten equation by nonlinear regression (GraphPad Prism v. 5.03) to determine K_M_ and V_max_ values (Figure S2).

### *Escherichia Coli* Growth Assays

*E. coli* TR61 cells containing various pBAD18-Kan:*AlaAT* or pBAD18-Kan:*GGAT* constructs were assayed for growth over 26 hrs. One mL of an overnight starter culture grown in LB medium was added to 100 mL of modified M63 minimal medium containing 0.2% glycerol, 0.2% arabinose, 50 µg mL^−1^ kanamycin and chloramphenicol at 25 µg mL^−1^. Bacterial cultures were then grown in flasks at 37°C for 26 hrs. After 4 hrs of growth all cultures were re-inoculated with 0.1% arabinose. The OD_600_ was recorded at 1–4 hr intervals through 14 hrs of growth and then again at 26 hrs for induced cultures, untransformed controls, empty vector controls, and uninduced controls containing a pBAD18-Kan:*HvAlaAT*.

TR61 cells containing an empty pBAD18-Kan vector as well as TR61 cells containing pBAD18-Kan:*HvAlaAT* were also assayed for growth differences in M63 liquid minimal medium, supplemented as described above, containing 2-oxoglutarate at different concentrations (Figure 2). Cell growth under these conditions was also assayed for a total of 26 hrs, with measurements taken every 1–3 hrs for the first 14 hrs and then again at 26 hrs.

## Supporting Information

Figure S1 **Amino acid sequence alignment for eleven AlaAT enzyme sequences and two GGAT enzymes sequences.** Amino acid sequences used were obtained from NCBI, except *M. truncatula* which was provided by Anis Limami, at the Université d' Angers, and analysis was done using ClustalW software. Residues conserved in subtype I aminotransferases are highlighted in white text on a black background. Fully conserved residues are indicated by “*”, conservative substitutions are indicated by “:”, and “.” denotes a semi-conservative substitution.
(TIF)

Figure S2 **K_M_ and V_max_ of various AlaAT enzymes with alanine, 2-oxoglutarate, pyruvate and glutamate.** Data were fitted to the Michaelis-Menten equation with the nonlinear regression facility of GraphPad Prism v. 5.03, in order to calculate K_M_ and V_max_ values. Data points are the mean ± standard error (SE) of triplicate determinations.
(TIF)

Table S1 **Primer sequences used in the cloning of AlaAT enzymes.** All *AlaATs* were cloned into the pBAD18-Kan plasmid using the restriction sites indicated. Restriction enzyme sites are shown in lower case lettering.
(TIF)

Table S2 **Average V_max_ values for unpurified AlaAT and GGAT enzymes.** V_max_ values are shown for each substrate, for each of the ten enzymes examined. Kinetic values represent the average of three independent trials. The correlation coefficient (r^2^) was >0.80 for all trials, except AtGGAT1 glutamate, AtGGAT2 glutamate and MmAlaAT1 pyruvate. Raw data are plotted in Figure S2.
(TIF)
