# Supplementary figures and images for: Analysis of the Enzymatic Properties of a Broad Family of Alanine Aminotransferases
Source: PLoS One. 2013 Feb 7;8(2):e55032. doi: 10.1371/journal.pone.0055032 (PMC3567105; doi:10.1371/journal.pone.0055032)

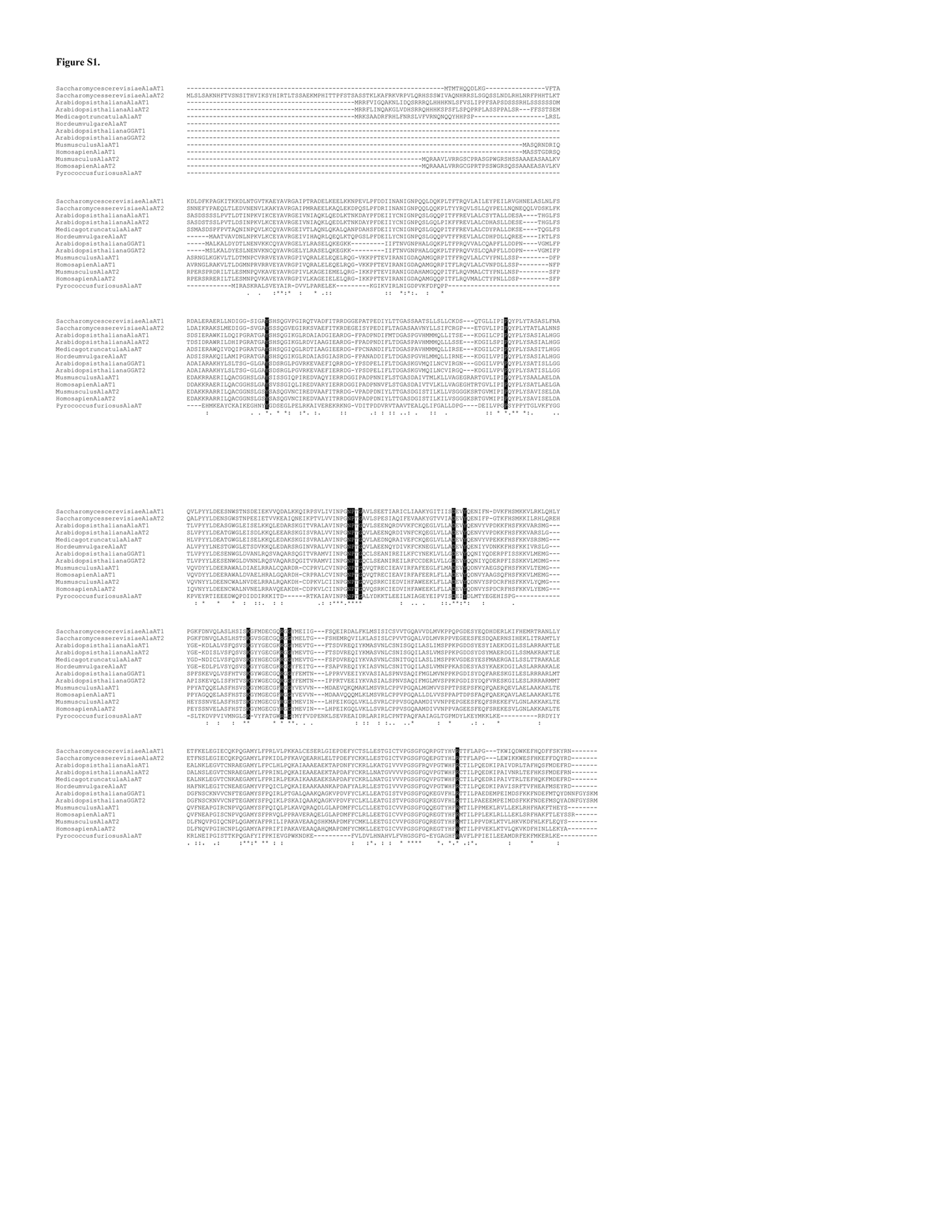

Supplement: Figure S1 — Amino acid sequence alignment for eleven AlaAT enzyme sequences and two GGAT enzymes sequences. Amino acid sequences used were obtained from NCBI, except M. truncatula which was provided by Anis Limami, at the Université d' Angers, and analysis was done using ClustalW software. Residues conserved in subtype I aminotransferases are highlighted in white text on a black background. Fully conserved residues are indicated by “*”, conservative substitutions are indicated by “:”, and “.” denotes a semi-conservative substitution. (TIF) [file pone.0055032.s001.tif]

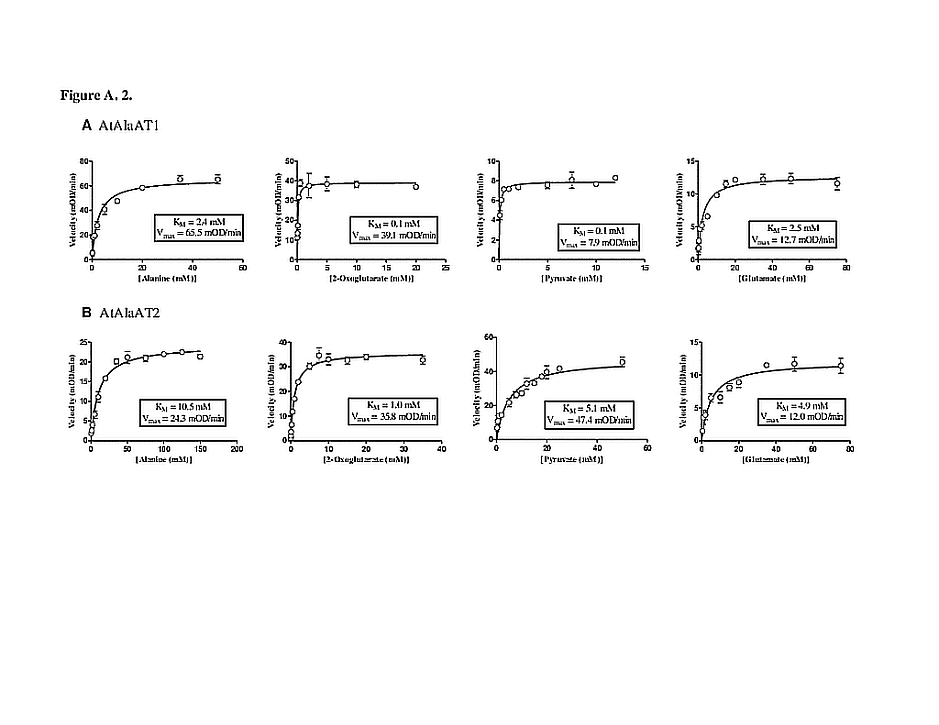

Supplement: Figure S2 — KM and Vmax of various AlaAT enzymes with alanine, 2-oxoglutarate, pyruvate and glutamate. Data were fitted to the Michaelis-Menten equation with the nonlinear regression facility of GraphPad Prism v. 5.03, in order to calculate KM and Vmax values. Data points are the mean ± standard error (SE) of triplicate determinations. (TIF) [file pone.0055032.s002.tif]

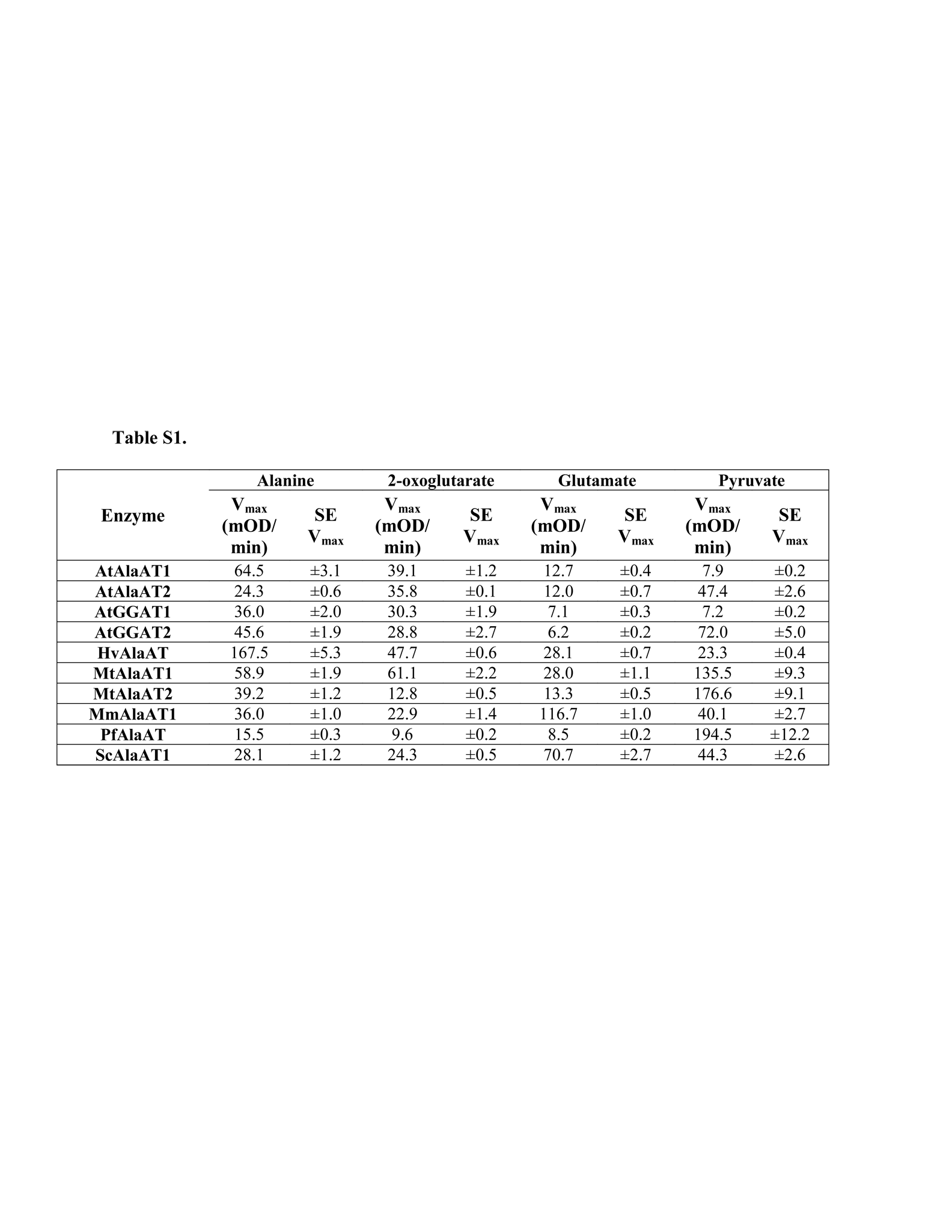

Supplement: Table S1 — Primer sequences used in the cloning of AlaAT enzymes. All AlaATs were cloned into the pBAD18-Kan plasmid using the restriction sites indicated. Restriction enzyme sites are shown in lower case lettering. (TIF) [file pone.0055032.s003.tif]

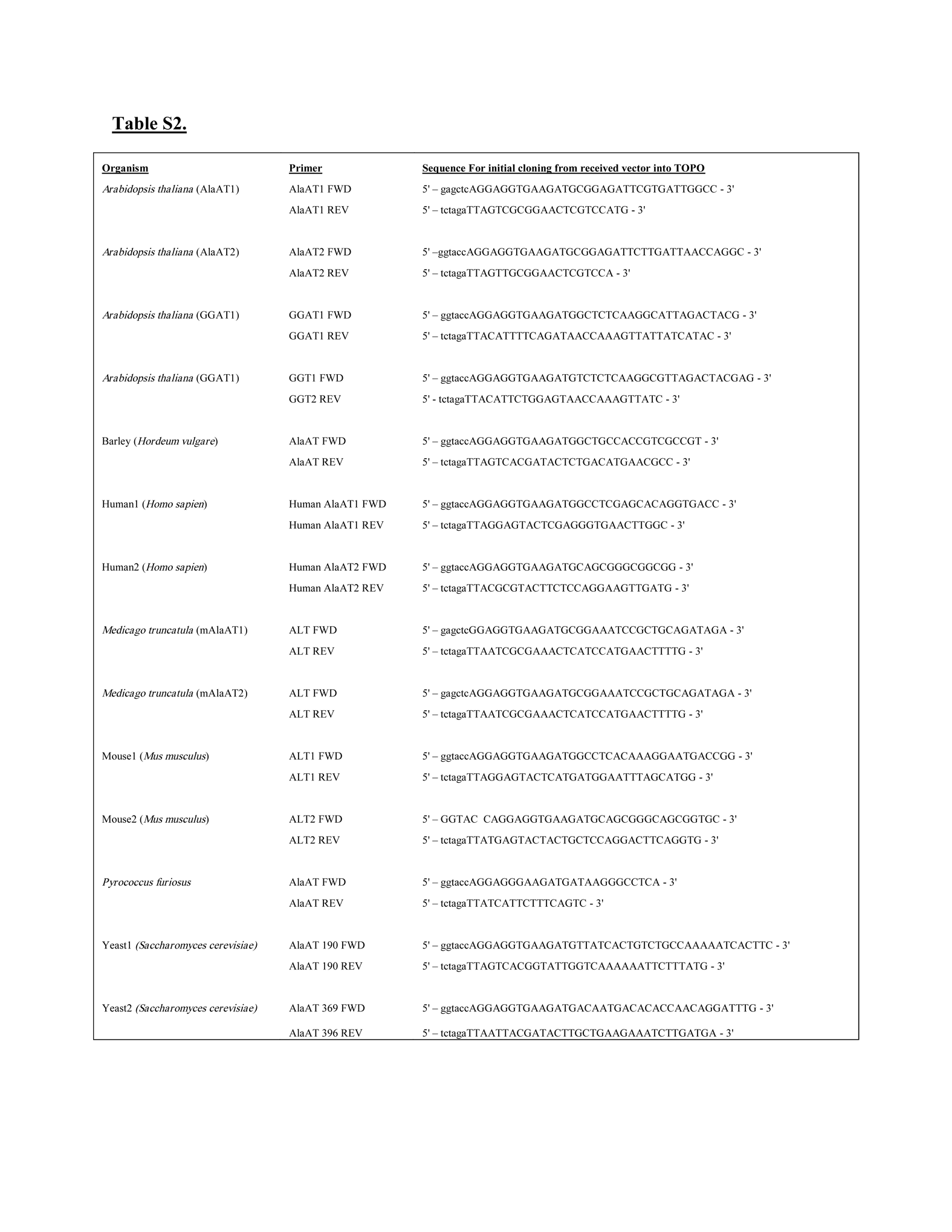

Supplement: Table S2 — Average Vmax values for unpurified AlaAT and GGAT enzymes. Vmax values are shown for each substrate, for each of the ten enzymes examined. Kinetic values represent the average of three independent trials. The correlation coefficient (r2) was >0.80 for all trials, except AtGGAT1 glutamate, AtGGAT2 glutamate and MmAlaAT1 pyruvate. Raw data are plotted in Figure S2. (TIF) [file pone.0055032.s004.tif]
